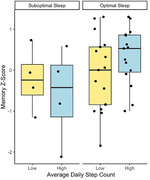# The Synergistic Effect of Sleep and Physical Activity on Memory in Cognitively Normal Older Adults

**DOI:** 10.1002/alz.089533

**Published:** 2025-01-09

**Authors:** Lana S. Callies, Coty Chen, Shannon Y. Lee, Valentina E. Diaz, Miwa Tucker, Savannah R. Hallgarth, Carina Lo, Anna M. VandeBunte, Natalie Pandher, Esther Li, Felicia Song, Quentin Coppola, Rowan Saloner, Caitlin Wei‐Ming Watson, Shubir Dutt, Thomas C. Neylan, Joel H. Kramer, Christine M Walsh, Kaitlin B. Casaletto, Emily W. Paolillo

**Affiliations:** ^1^ Memory and Aging Center, UCSF Weill Institute for Neurosciences, University of California, San Francisco, San Francisco, CA USA; ^2^ University of Florida, Gainesville, FL USA; ^3^ Northeastern University, Boston, MA USA; ^4^ Memory and Aging Center, UCSF Weill Institute for Neurosciences, University of California San Francisco, San Francisco, CA USA

## Abstract

**Background:**

Modifiable risk factors are important for prevention of age‐related cognitive decline. Prior research has linked both physical activity (PA) and sleep with better memory outcomes. To better understand their potential synergistic effects, we examined independent and interactive effects of actigraphy‐based PA and total sleep time on cognitive functioning in cognitively unimpaired older adults.

**Method:**

Participants included 40 cognitively unimpaired older adults (age = 71.1 ± 10.8yrs; 52% female; 75% White; mean education = 18.2yrs) enrolled in studies at the UCSF Memory and Aging Center. All participants completed in‐person neuropsychological assessments and 30 days of Fitbit^TM^ Inspire 2 monitoring, which tracked average nightly total sleep time and average daily step count. Average sleep time was dichotomized into optimal sleep (6‐8hrs) and sub‐optimal sleep (<6hrs, >8hr) based on previous literature, and average daily step count was dichotomized by median split (high PA ³ 8490 steps). Composite cognitive z‐scores were calculated for memory and executive functioning. Multiple linear regression models examined the interaction between sleep and PA on cognitive domain z‐scores, controlling for age, sex, and education.

**Result:**

Neither sleep nor average daily steps were independently related to memory (sleep:*b* = ‐0.58, 95%CI [‐1.36, 0.20], *p* = 0.141; steps: *b* = ‐0.27, 95%CI [‐0.86, 0.32], *p* = 0.357) or executive functioning (sleep:*b* = ‐0.68, 95%CI [‐1.46, 0.10], *p* = 0.087; steps: *b* = ‐0.21, 95%CI [‐0.82, 0.40], *p* = 0.487). In combination, however, sleep significantly moderated the effect of PA on memory (*b* = 1.47, 95%CI [0.03, 2.92], *p* = 0.046) such that higher PA related to better memory performance only among participants with optimal sleep times. The interaction between PA and sleep on executive functioning was not significant (*b* = 0.49, 95%CI [‐1.06, 2.03], *p* = 0.528).

**Conclusion:**

Using unobtrusive objective monitoring of modifiable lifestyle factors via wearable actigraphy, we found a beneficial synergistic relationship between sleep and PA on memory. Results highlight the clinical relevance of supporting multi‐domain improvements in modifiable lifestyle factors, including optimal sleep time and higher PA in older adults.